# Significant relationship between parameters measured by transrectal color Doppler ultrasound and sexual dysfunction in patients with BPH 12 months after TURP

**DOI:** 10.1186/s12894-020-00776-2

**Published:** 2021-01-13

**Authors:** Li K. Chen, Yu W. Lai, Li P. Chiu, Saint Shiou-Sheng Chen

**Affiliations:** 1 Department of Anesthesiology, China Medical University, Taichung City, Taiwan; 2 Department of Anesthesiology, China Medical University Hospital, Taichung City, Taiwan; 3 Division of Urology, Taipei City Hospital Ren Ai Branch, Taipei, Taiwan; 4 Department of Urology, National Yang-Ming University School of Medicine, Taipei, Taiwan; 5 Division of Urology, Taipei City Hospital Chushing Branch, Taipei, Taiwan; 6 General Education Center, University of Taipei, Taipei, Taiwan; 7 Division of Urology, Taipei City Hospital Zhong Xiao Branch, Taipei, Taiwan; 8 Commission for General Education, College of Applied Science, National Taiwan University of Science and Technology, Taipei, Taiwan

**Keywords:** Benign prostatic hyperplasia, Erectile dysfunction, Sexual dysfunction, Transrectal color doppler ultrasound, transurethral resection of the prostate

## Abstract

**Background:**

A link between sexual dysfunction and lower urinary tract symptoms due to benign prostatic hyperplasia (BPH) has been noticed. Transurethral resection of the prostate (TURP) remains the standard treatment for symptomatic BPH, whether TURP causes sexual dysfunction is still uncertain. In this retrospective study, we investigated the relationship between parameters measured by color Doppler ultrasound (CDU) and sexual dysfunction in patients with BPH 12 months after TURP.

**Methods:**

The parameters include presumed circle area ratio (PCAR), maximal horizontal area of seminal vesicles (MHA), resistive index of the prostate (RIP), and peak systolic velocity in the flaccid penis (PSV). The international prostate symptom score was used to evaluate the lower urinary tract symptoms and the five-item version of the International Index of Erectile Function was used to evaluate sexual function before and after TURP.

**Results:**

Of the 103 patients without sexual dysfunction before TURP, 11 (10.7%) had erectile dysfunction (ED) after TURP. These 11 patients had significantly lower PCAR, RIP, PSV and MHA than those without ED. The patients with retrograde ejaculation after TURP had significantly lower PCAR than those without retrograde ejaculation, and the patients with premature ejaculation after TURP had significantly lower MHA than those without premature ejaculation. Comparing the parameters between baseline and after TURP, PCAR, RIP, and MHA decreased significantly in the patients with sexual dysfunction, but no significant differences were noted in the patients without sexual dysfunction after TURP.

**Conclusions:**

More extended TURP can lead to a higher incidence of ED and retrograde ejaculation in BPH patients without sexual dysfunction before TURP. Patients with a lower volume of seminal vesicles after TURP may have a higher incidence of premature ejaculation.

## Background

A link between sexual dysfunction and lower urinary tract symptoms (LUTS) due to benign prostatic hyperplasia (BPH) has been reported [1]. Sexual dysfunction includes erectile dysfunction (ED) and ejaculatory dysfunction, and ejaculatory dysfunction includes premature ejaculation, retrograde ejaculation, delayed ejaculation, painful ejaculation, and decreased force of ejaculation [1]. Transurethral resection of the prostate (TURP) remains the standard treatment for symptomatic BPH, which occurs in about 10% to 20% of men over 40 years of age [2–4]. Some patients may experience complications of TURP including TURP syndrome, bladder neck contracture (BNC), bleeding, incontinence, ED and ejaculatory dysfunction, some of which have been reported to be associated with prostate size and surgical time [2, 5]. However, Pavone et al. suggested that TURP did not have a negative impact on ED in contrast to ejaculatory dysfunction [6]. In addition, Muntener et al. reported that TURP did not have a negative influence on sexual activity [7]. Therefore, whether TURP causes sexual dysfunction is still uncertain.

Many methods have been used to measure prostate size, including a digital rectal examination (DRE), cystourethroscopy and retrograde urethrography, however the results of these modalities are poor compared to transrectal ultrasound [8, 9]. Watanabe et al. reported good results using transrectal ultrasound to measure prostate volume [10]. Presumed circle area ratio (PCAR) has been shown to be correlated with LUTS, and especially obstructive symptoms [11, 12]. Changes in prostate size on transrectal ultrasound after TURP for patients with BPH have been reported [13], and our previous study showed a reduction in prostate volume 12 weeks after TURP [14]. Nevertheless, the clinical significance of such changes on transrectal ultrasound is still unclear.

Few studies have assessed the correlation between changes in prostate and seminal vesicle size and blood flow in the prostate and penis after TURP and sexual dysfunction. We hypothesize that TURP will cause morphological change in the prostatic fossa which may compromise the blood flow in the prostate, seminal vesicle and penis and result in sexual dysfunction. Therefore, we conducted this retrospective study by using parameters such as PCAR, resistive index in the prostate (RIP), and maximal horizontal area of seminal vesicles (MHA) measured by transrectal color Doppler ultrasound (TRUS); and peak systolic velocity in the flaccid penis (PSV) measured by color Doppler ultrasound (CDU) to evaluate the possible mechanism of sexual dysfunction after TURP in BPH patients without sexual dysfunction before TURP.

## Methods

### Patients

The Institutional Review Board of Taipei City Hospital approved this study (TCHIRB-10506107-E). Between August 2004 and November 2010, 154 men with symptomatic BPH who received TURP were evaluated. All the patients received α-blocker treatment and no patients took 5-α-reductase inhibitor or phosphodiesterase-5 inhibitors before TURP. They agreed to provide regular follow-up including TRUS and CDU of flaccid penis for at least one year following written informed consent. In this period, we used a monopolar cutting loop and 5% glucose water as irrigating fluid to perform TURP, including in diabetic patients who had blood sugar monitored after surgery. Two experienced urologists preformed TURP and removed all the prostate tissue completely until they visualized the surgical capsule of the prostate. Fifty one patients were excluded including 8 did not have complete data, 38 had ED and 5 had poorly controlled diabetes mellitus (DM, fasting blood sugar > 300 or HbA1c > 8.0) before TURP. The remaining 103 patients were included for evaluation, and they were sexually active and did not have sexual dysfunction including ED or ejaculatory dysfunction before medical treatment or TURP, and they all wanted to maintain sexual function after TURP.

### Measurement of transrectal color Doppler ultrasound (TRUS) and color Doppler ultrasound (CDU)

TRUS and CDU were performed in all patients using a real-time scanner with a rotating 7.5 MHz transducer (Bruel & Kjaer, Copenhagen, Denmark) 1 month before and 12 months after TURP. Two surgeons performed all of the procedures and ultrasound examinations. PCAR was defined as the ratio of the area of the maximum horizontal section of the prostate measured by TRUS to the area of a presumed circle of which the circumference was equal to the circumference of the maximum horizontal section (to calculate how closely the section approaches a circle), and a PCAR of 1.0 reveals that the section is equal to a circle [11, 12]. MHA was defined as the maximal horizontal area of seminal vesicles by TRUS, and was calculated as maximal length x maximal width of the seminal vesicle. We routinely evaluate seminal vesicles during TRUS at our hospital. RIP (peak systolic velocity—end diastolic velocity/peak systolic velocity) was used to detect blood flow in the prostate by TRUS. Peak systolic velocity in the flaccid penis (PSV) was measured by CDU one month before and 12 months after TURP. The international prostate symptom score (IPSS) was used to evaluate the lower urinary tract symptoms (LUTS), and the five-item version of the International Index of Erectile Function (IIEF-5) was used to evaluate sexual function one month before and 12 months after TURP in all of the patients. Retrograde ejaculation was defied as no semen was found when ejaculated and premature ejaculation was defined as you almost ejaculated before you wished and were very difficult to delay ejaculation 12 months after TURP according to the record of chart.

### Statistical analysis

The Mann–Whitney U test was used for statistical analysis, and a P value < 0.05 was regarded to indicate statistical significance. The Mann–Whitney U test is a nonparametric test of the null hypothesis. It does not require the assumption of normal distributions and is nearly as efficient as the t-test on normal distributions. IBM SPSS Statistics for Windows, Version 20.0 (Armonk, NY: IBM Corp.) was used for the statistical analysis.

## Results

The total number of TURP procedures performed during this period was 1205. The mean prostate volume was 68.5 cm^3^ and the median was 67.5 cm^3^, and the mean resected weight was 52.5 g and the median was 51.1 g. The mean operative time was 90.5 min. Of the 103 patients (mean age 70.3 years, range 54–85 years) without sexual dysfunction before TURP (IIEF-5: 25.6 ± 3.0), 11 (10.7%) complained of ED (IIEF-5: 10.2 ± 3.1, after TURP), and the IIEF for the other 92 patients was 25.1 ± 3.0 after TURP. Of the 103 patients, seven had DM, of whom two (28.6%) had ED after TURP. Capsular perforation was found in eight patients, of whom four (50.0%) had ED. Capsular perforation was determined by the surgeon through visual inspection during TURP and recorded in the operation note. The patients with ED after TURP had higher incidence rates of DM and capsular perforation than those without ED. Of the eight patients with BNC after TURP, two (25%) had ED, which was higher than the total incidence (10.7%).

Patients with BPH who had ED after TURP had significantly lower PCAR, RIP, MHA and PSV than those who did not have ED after TURP (Table 1). There was no significant difference in age between the patients with or without ED after TURP (Table 1). Of the 103 patients, 58 (56.3%) had retrograde ejaculation and six had premature ejaculation (5.8%) after TURP. All the 64 patients with ejaculatory dysfunction after TURP did not have ED. The patients with retrograde ejaculation after TURP had significantly higher PCAR than those without (Table 1); however there were no significant differences in age, RIP, MHA and PSV. The patients with premature ejaculation after TURP had lower MHA than those without premature ejaculation (Table 1). There were no significant differences in age, PCAR, RIP and PSV between the patients with or without premature ejaculation after TURP.

**Table 1:** PCAR, RIP, MHA, PSV, IPSS and IIEF-5 before and 12 months after TURP in difference age groups with respect to sexual dysfunction

	ED (+), N = 11	ED (−), N = 92	*p*
PCAR^b^ PCAR^a^ *p* RIP (ml/s)^b^	0.89 ± 0.07 0.67 ± 0.04 0.02* 0.81 ± 0.05	0.87 ± 0.08 0.83 ± 0.06 0.19 0.84 ± 0.08	0.31 0.03* 0.21
RIP (ml/s)^a^ *p* MHA (cm^2^)^b^	0.59 ± 0.04 0.01* 7.8 ± 1.8	0.77 ± 0.08 0.06 8.0 ± 2.2	0.02* 0.21
MHA (cm^2^)^a^ *p* PSV (cm/s)^b^ PSV (cm/s)^a^ *p* IPSS^b^ IPSS^a^ *p*	4.7 ± 1.5 0.01* 14.3 ± 1.9 9.1 ± 1.6 0.01* 20.9 ± 4.6 2.6 ± 0.7 0.01*	7.5 ± 2.3 0.09 14.7 ± 2.0 14.4 ± 2.1 0.27 19.6 ± 4.2 2.9 ± 0.8 0.01*	0.01* 0.27 0.01* 0.27 0.08
IIEF-5^b^ IIEF−5^a^ *p*	25.9 ± 3.1 10.2 ± 3.1 0.01*	25.6 ± 2.9 25.1 ± 3.0 0.29	0.26 0.01*
Age (years)	71.5 ± 8.1	68.8 ± 8.7	0.06
PV (cm^3^)	69.1 ± 10.5	68.4 ± 10.2	0.15
RW (gm)	52.9 ± 8.2	52.5 ± 7.9	0.22

	Retrograde ejaculation ( +), N = 58	Retrograde ejaculation (−), N = 45	*p*
PCAR^b^ PCAR^a^ *p* RIP (ml/s)^b^	0.88 ± 0.09 0.79 ± 0.05 0.04* 0.82 ± 0.08	0.86 ± 0.07 0.84 ± 0.06 0.21 0.86 ± 0.11	0.21 0.04* 0.11
RIP (ml/s)^a^ *p* MHA (cm^2^)^b^	0.70 ± 0.08 0.04* 8.2 ± 2.2	0.80 ± 0.11 0.06 7.7 ± 1.8	0.07 0.07
MHA (cm^2^)^a^ *p* PSV (cm/s)^b^ PSV (cm/s)^a^ *p* IPSS^b^ IPSS^a^ *P*	7.1 ± 2.1 0.04* 14.1 ± 1.9 13.1 ± 2.2 0.07 19.9 ± 4.1 2.6 ± 0.5 0.01*	7.3 ± 1.9 0.07 15.3 ± 2.3 14.8 ± 2.1 0.08 19.5 ± 4.4 3.2 ± 1.2 0.01*	0.15 0.15 0.14 0.31 0.07
IIEF-5^b^ IIEF-5^a^ *P*	25.8 ± 3.0 23.1 ± 2.4 0.18	25.3 ± 2.8 24.0 ± 2.7 0.19	0.26 0.22
Age (years)	69.8 ± 9.3	70.1 ± 9.7	0.36
PV (cm^3^)	68.6 ± 10.3	68.3 ± 10.1	0.26
RW (gm)	52.7 ± 8.0	52.2 ± 7.8	0.24

	Premature ejaculation ( +), N = 6	Premature ejaculation (−), N = 97	*p*
PCAR^b^ PCAR^a^ *p* RIP (ml/s)^b^	0.88 ± 0.08 0.80 ± 0.05 0.04* 0.83 ± 0.09	0.87 ± 0.07 0.81 ± 0.06 0.06 0.84 ± 0.11	0.28 0.13 0.21
RIP (ml/s)^a^ *p* MHA (cm^2^)^b^	0.74 ± 0.09 0.03* 7.9 ± 1.3	0.77 ± 0.09 0.06 8.0 ± 2.4	0.09 0.21
MHA (cm^2^)^a^ *p* PSV (cm/s)^b^ PSV (cm/s)^a^ *p*	4.1 ± 1.3 0.02* 15.1 ± 2.4 12.9 ± 1.7 0.06	7.7 ± 2.4 0.09 14.6 ± 2.0 13.9 ± 1.9 0.07	0.01* 0.19 0.09
IPSS^b^	19.5 ± 4.0	19.8 ± 4.3	0.31
IPSS^a^ *p*	2.7 ± 0.8 0.01*	2.9 ± 0.8 0.01*	0.11
IIEF-5^b^ IIEF-5^a^ *p*	25.7 ± 3.0 20.9 ± 2.6 0.06	25.6 ± 2.9 23.7 ± 2.7 0.14	0.28 0.19
Age (years)	70.3 ± 9.9	70.0 ± 8.9	0.38
PV(cm^3^)	68.8 ± 10.4	68.5 ± 10.2	0.29
RW (gm)	52.8 ± 8.1	52.5 ± 7.8	0.31

PCAR: presumed circle area ratio of the prostate; RIP: resistive index in the prostate; MHA: maximal horizontal area of seminal vesicles; PSV: peak systolic velocity in the flaccid penis; IPSS: international prostate symptom score; IIEF-5: the five-item version of the International Index of Erectile Function; PV: prostate volume; RW: resected prostatic weight; N: patient number; ^b^: before TURP; ^a^:12 months after TURP; *p < 0.05 indicates a significant difference; Data are expressed as mean ± SD; The Mann–Whitney U test was used for statistical analysis.

There were no significant differences in PCAR, RIP, MHA, PSV and age before TURP between the patients with or without ED or ejaculatory dysfunction after TURP (Table 1). Comparing the parameters between baseline and after TURP, PCAR, RIP, and MHA decreased significantly in the patients with sexual dysfunction, but no significant differences were noted in the patients without sexual dysfunction after TURP (Table 1). PSV decreased significantly in the patients with ED after TURP, but no significant difference was noted in the patients with ejaculatory dysfunction after TURP (Table 1). LUTS improved in all of the patients 12 months after TURP (IPSS: 19.7 ± 4.2 before TURP vs. 2.9 ± 0.8 after TURP). Patients who had sexual dysfunction after TURP had a lower IPSS than those without sexual dysfunction, but the difference was not statistically significant (Table 1).

## Discussion

Ten to 20% of men over 40 years of age have LUTS due to BPH, and TURP is currently the treatment of choice for these patients with a success rate of 85 to 90% [2–4]. Estimating the prostate volume before surgery is important because most patients with BPH are older and may not be able to tolerate a prolonged operation. However, a DRE, retrograde urethrography, urethrocystoscopy and urethral pressure profile may not accurately estimate prostate volume, especially for moderate and large prostates [10, 15]. Tewari et al. reported that transrectal ultrasound more accurate in estimating prostate volume than MRI [16], and Terris et al. suggested that the prolate spheroid formula could most accurately estimate the prostate weight, especially for a prostate of < 80 g [17]. In this study, we used the Eq. (0.52 × L x W x H) to measure prostate volume and calculate PCAR.

The principle of electrosurgery was evaluated by Mclean [18]. A conventional TURP resectoscope uses an electric current with a cutting loop to cut the prostatic adenoma, and bleeding is controlled by cauterization. However, some complications may occur, including BNC, ED, incontinence and ejaculatory dysfunction, and the reported rate of ED after TURP is about 3.5 to 8.3% [3, 5, 19–23]. Favilla et al. reported that patients older than 65 years had a higher risk of developing ED after TURP [24]. Corona et al. suggested that PSV (< 13 cm/s) measured in the flaccid penis by CDU can be used to diagnose ED with an accuracy higher than 80% [25]. Therefore, we used PSV in this study to detect penis blood flow. However, other reports have suggested that TURP does not have a negative influence on erectile function [6, 7]. Tuncel et al*.* found RI of neurovascular bundle decreased in patients with ED after prostate biopsy and suggested ED following biopsy of prostate might have an organic basis [26]. In this study, the ED rate was 10.7% after TURP. Furthermore, we demonstrated that the patients who had ED after TURP had significantly lower PCAR, RIP, MHA and PSV than those without ED. In addition, the patients with ED also had significantly lower PCAR, RIP, MHA and PSV after TURP compared to baseline. A possible explanation is that more extended TURP may cause lower PCAR, and reduce the blood flow in the prostate and penis, which may then cause ED. Tuncel et al. and Zisman et al*.* suggested prostate biopsy induced ED may happen by direct damage to neurovascular bundle or secondary trauma to nerve by compression of hematoma or edema [26, 27]. Patients with lower PCAR after more extended TURP may lead to more indirect nerve injury; especially all the patients underwent monopolar TURP in this study. Reductions in RIP and MHA after TURP may be a consequence of low sexual activity due to ED rather than the co-effect of ED; however all of the patients in this study wanted to be sexually active after TURP. Although no significant difference in age was noted in this study even in patients > 70 years compared with those < 70 years, further studies are needed to clarify this issue. It would have been better to assess psychological impact of surgery on ED (libido/IIEF-15 questionnaire) as patients included in the study had wide range of age (54–85 years).

Ejaculation is a complicated process involving the emission and expulsion of semen, and includes somatic, sympathetic and parasympathetic pathways, and factors which interfere the balance between sympathetic and parasympathetic nerves can induce ejaculatory dysfunction [28]. Retrograde ejaculation is permitted by a pathologically open internal vesical sphincter or bladder neck and painful ejaculation may be induced by an abnormal sensation and inflammation of the prostate [1, 29]. Ejaculatory dysfunction has been reported in 65% of patients after TURP, with a retrograde ejaculation rate of about 50% after TURP [1, 24]. Bladder neck resection during TURP may lead to retrograde ejaculation and we routinely preformed this procedure for every patient in this study. The incidence of retrograde ejaculation in this study was 56.3%, which is higher than in other reports, and we first noticed that it occurred more frequently in the patients with lower PCAR after TURP. This may be because the wider prostatic fossa after TURP may decrease resistance of the bladder neck and increase the rate of retrograde ejaculation. We hypothesize that decreased blood flow in the prostate and neurovascular bundle may compromise the contractility of ejaculation-related muscles, and therefore greater contractility will be needed which may cause retrograde ejaculation. Side effect of α-blocker treatment is retrograde ejaculation, but no patients receive α-blocker after TURP in this study. However, further studies are needed to clarify the mechanism. The incidence of premature ejaculation after TURP is low and the incidence is 5.8% in this study. Patients who had premature ejaculation had lower MHA than those without premature ejaculation and a possible explanation is that decreased volume of seminal vesicle after TURP might increase the pressure and sensitivity of seminal vesicle which may induce premature ejaculation. MHA can change before and after ejaculation and a volume of seminal vesicle might be influenced by the timing of ejaculation, but we did not consider ejaculation timing in this study. The impact of blood flow in the penis on ejaculatory dysfunction is unclear. The patients with ejaculatory dysfunction had lower PSV after TURP than those without in this study. However, more studies are needed to evaluate these issues.

Wasson et al. concluded that more older men are now receiving TURP compared to the 1980s, and that the outcomes for men older than 65 years are good [30]. Symptoms of moderate to severe ejaculatory dysfunction increase with age [1, 31]. A possible explanation is that the older patients may have had a lower testosterone level, lower volume of semen and lower contractility of ejaculation-related muscles, which may have become more severe after TURP. However, in this study, there was no significant difference of age between patients with ejaculatory dysfunction or without ejaculatory dysfunction after TURP, which was different from the previous studies. Therefore, further studies are needed to evaluate these complicated issues.

We found that the patients who complained of sexual dysfunction 12 months after TURP had better surgical outcomes than those without sexual dysfunction (lower IPSS after TURP); however the difference was not statistically significant. The reason might be that more extended TURP will improve LUTS, but will induce a higher incidence of sexual dysfunction. However, more studies are needed to evaluate this issue. The limitations of this study are as follows. First: this is a retrospective study and some patients were excluded because they had ED before TURP or incomplete data. Second: the case number is relatively small. Third: there was no specific control group for comparison. Fourth: TURP was performed by different urologists, and so inter-operator bias might exist. Fifth: none of the patients received bipolar or laser TURP in this study. Sixth: the diagnosis of sexual dysfunction was according to the IIEF-5 scores and chart record.

## Conclusions

More extended TURP may cause lower PCAR, RIP, MHA and PSV, which may compromise the blood supply in the penis and prostate, then induce a higher incidence of ED or retrograde ejaculation in BPH patients without ED 12 months after TURP. Patients with a lower volume of seminal vesicles 12 months after TURP may have a higher incidence of premature ejaculation.

## Data Availability

The data and materials were collected and analyzed by Dr. LKC, Dr. YWL and Miss LPC. I assure that the article is original. It is not under consideration for publication by another journal and the data have never been published previously. The datasets used and/or analysed during the current study available from the corresponding author on reasonable request.

## Abbreviations

BPH: Benign prostatic hyperplasia
TURP: Transurethral resection of the prostate
CDU: Color Doppler ultrasound (CDU)
PCAR: Presumed circle area ratio
MHA: Maximal horizontal area of seminal vesicles
RIP: Resistive index of the prostate
PSV: Peak systolic velocity in the flaccid penis
IPSS: The international prostate symptom score
LUTS: Lower urinary tract symptoms
IIEF-5: The five-item version of the International Index of Erectile Function
ED: Erectile dysfunction
